# Practical Notes on Diagnosis and Treatment

**Published:** 1907-03-02

**Authors:** 


### Practical Notes on Diagnosis and Treatment.

### Bone Marrow in Pernicious Anæmia.

The red marrow may be taken direct from a long bone of the ox in teaspoonful doses with a little salt, or it may be made into sandwiches. It may also be obtained in tablet form or in a solution in glycerine. But the fresh marrow is to be preferred. *Dr. W. Miller Ord.*

### Senile Pruritus.

Many cases of senile pruritus to which no cause can be assigned beyond atrophy of the skin, can be relieved by tincture of cannabis indica, beginning with 5 and increasing to 20 or even 30 minims thrice daily. The remedy should be well diluted and taken after meals. Another useful agent is pilocarpine, which should be given by subcutaneous injection and may be continued for weeks or months. —*Dr. H. G. Adamson.*

### Ethyl Chloride.

Ethyl chloride is the best anæsthetic known for single-dose cases—namely, those in which anæsthesia is induced, the mask removed, and the operation performed without any further administration. This is more particularly true of children. In operations for enlarged tonsils and adenoids ethyl chloride is destined to save many lives. The chloroform mortality in these operations is exceptionally high, over a hundred deaths occurring in these circumstances in Great Britain between 1890 and 1900.—*Mr. J. H. Chaldecott.*

### Oatmeal in Diabetes.

The oat cure consists in the daily administration of 200 to 250 grammes of oatmeal, best given in the form of gruel every two hours; 200 to 300 grammes of butter and often about 100 grammes of vegetable proteid, or a few eggs may be taken in addition. Nothing else is allowed except black coffee at tea, lemon juice, good old wine, or a little brandy or whisky. At first there may be increase of the glycosuria, but after a few days the sugar diminishes and the acetonuria still more so. The treatment has been found to give the best result in severe cases.—*Professor Carl von Noorden.*

### Bulbar Paralysis.

Difficulty of articulation and dribbling of the saliva are prominent features in bulbar paralysis. Hence it has been said: "When you see a patient come into the room with a slate in one hand and a handkerchief in the other, you may say 'bulbar paralysis' at once."

### Prognosis in Mitral Stenosis.

In many instances mitral stenosis is a condition which causes the patient but little disturbance, even over a term of years, and whilst it necessarily introduces certain risks and dangers, its existence does not invariably demand the grave prognosis often attached to it.—*Sir Wm. Gairdner.*

### Treatment of Dysmenorrhœa.

In congestive dysmenorrhœa the pain does not yield readily to antispasmodics but will sometimes yield promptly to extract of cannabis indica ($\frac{1}{3}$ grain) or to a combination of hydrastis, belladonna, and bromide of potassium or sodium. Efforts should also be made to promote the menstrual flow and to diminish congestion by acting on the bowels, kidneys and skin. . . . In cases of pelvic congestion with cold extremities and cold skin over the body, nitro-glycerine is remarkably effective.—*Dr. Amand Routh.*

### Treatment of Eclampsia.

At once administer a purgative, say, 5 grains of calomel with a drachm of compound jalap powder if the patient is conscious; if she is unconscious, give two minims of croton oil by the œsophageal tube and follow this by an enema. Promote the action of the skin by copious draughts of fluid and by a hot pack or hot air bath; a pint of saline fluid may also be injected subcutaneously into each axilla. Give morphine—$\frac{1}{2}$ grain followed by $\frac{1}{4}$ grain doses if the fit persists. If the patient is in labour empty the uterus as soon as possible. If she is not yet in labour some advocate the above treatment alone, whilst others suggest forcible dilatation and delivery, or vaginal Cæsarean section.—*Mr. Bellingham Smith.*

